# Incomplete circle of Willis as a risk factor for intraoperative ischemic events during carotid endarterectomies performed under regional anesthesia – A prospective case-series

**DOI:** 10.1515/tnsci-2022-0293

**Published:** 2023-07-10

**Authors:** Zoltán Gyöngyösi, Ivett Belán, Edit Nagy, Zsófia Fülesdi, Orsolya Farkas, Tamás Végh, Arjan Willem Hoksbergen, Béla Fülesdi

**Affiliations:** Department of Anesthesiology and Intensive Care, University of Debrecen, Debrecen, Hungary; Department of Radiology, University of Debrecen, Debrecen, Hungary; Department of Vascular Surgery, Academic Medical Center Amsterdam, Amsterdam, The Netherlands

**Keywords:** carotid endarterectomy, circle of Willis, collateral ability, cross-clamping, local anesthesia

## Abstract

**Background:**

The role of the willisian collaterals during carotid endarterectomies (CEAs) is a debated issue. The aim of the present work was to test whether an incomplete or non-functional circle of Willis (CoW) is a risk factor for ischemic events during CEA.

**Patients and methods:**

CEAs were performed under local anesthesia. Patients were considered symptomatic (SY) if neurological signs appeared after the cross-clamping phase. In SY patients shunt insertion was performed. CoW on CT angiograms (CTa) were analyzed offline and categorized as non-functional (missing or hypoplastic collaterals) or functional collaterals by three neuroradiologists. Near-infrared spectroscopy (NIRS) was performed throughout the procedure.

**Results:**

Based on CTa, 67 incomplete circles were found, 54 were asymptomatic (ASY) and 13 were SY. No complete CoW was found among the SY patients. Significant differences could be detected between incomplete and complete circles between ASY and SY groups (Chi-square: 6.08; *p* = 0.013). The anterior communicating artery was missing or hypoplastic in 5/13 SY cases. There were no cases of the non-functional anterior communicating arteries in the ASY group (Chi-square: 32.9; *p* = 10^−8^). A missing or non-functional bilateral posterior communicating artery was observed in 9/13 SY and in 9/81 ASY patients (Chi-square: 24.4; *p* = 10^−7^). NIRS had a sensitivity of 76.9% and a specificity of 74.5% in detecting neurological symptoms.

**Conclusions:**

Collateral ability of the CoW may be a risk factor for ischemic events during CEAs. Further studies should delineate whether the preoperative assessment of collateral capacity may be useful in decision-making about shunt use during CEA.

Carotid endarterectomy (CEA) is an effective method for preventing ischemic strokes in patients having hemodynamically significant internal carotid artery stenosis. The surgical procedure requires the temporary interruption of the blood flow through the internal carotid artery in the ipsilateral hemisphere, eventually leading to neurological complications [1–3]. Cross-clamping of the common carotid artery during the procedure requires effective flow through the intracranial collaterals in order to maintain blood flow in the ipsilateral brain tissue. The most important first-line collateral pathway to ensure “backup” flow during this period is the circle of Willis (CoW).

However, several reports indicated that even in the non-cerebrovascular cohort the typical pattern of the willisian polygon is observed in 4.6–72.2% of the cases and variations as well as hypoplastic arterial segments may lead to non-functional collateral ability of the CoW [4–6]. Clinical studies pointed out that there is an association between the occurrence of ischemic strokes and collateral functionality of the CoW both in the cerebrovascular [7,8] and in the non-cerebrovascular [9] cohort. In an analysis of the North American Symptomatic Endarterectomy Trial group the presence of effective collaterals decreased the risk of ischemic events both in the medically treated and in the operated groups in patients with hemodynamically significant carotid artery stenosis [10]. The cross-clamping phase during CEA may be considered as an iatrogenically induced acute internal carotid artery occlusion that challenges the cerebral blood flow in the ipsilateral hemisphere, depending on the collateral capacity of the CoW. Monitoring opportunities during general anesthesia have limited sensitivity to detect cerebral ischemia and thus to indicate the insertion of a shunt as a preventive measure [11,12]. Therefore, there is an ongoing search for an accurate method that may preoperatively delineate patients at higher risk for a need of shunt insertion after carotid cross-clamp. Recently, the incomplete CoW has been shown to increase the risk of neurologic events in patients undergoing CEA without shunting [13,14]. In line with this, in the present study, we attempted to assess the relationship between the occurrence of neurological symptoms referring to ipsilateral ischemia and the collateral capacity of the CoW in patients undergoing CEA under general anesthesia. Our intention was to answer the following study questions:

1. What is the incidence of incomplete CoW in our cohort?
2. Is there a relationship between an incomplete CoW and the appearance of ischemic symptoms after cross-clamping?
3. How did brain tissue oxygen saturation measured by near-infrared spectroscopy (NIRS) reflect the clinical symptoms in asymptomatic (ASY) and symptomatic (SY) patients?

## 1 Patients and methods

This is a prospective case series that was performed in patients with unilateral hemodynamically significant carotid stenosis scheduled for elective endarterectomies at the Department of Surgery University of Debrecen.

Local anesthesia was performed under ultrasound guidance using the L12-4s linear probe of the TE7 Ultrasound System (Shenzhen Mindray Bio-Medical Electronics Co., Ltd, Nanshan, Shenzhen, China). A 22GA, 50 mm long regional block needle (Vygon Echoplex, Ecouen, France) was used. An intermediate and a superficial plexus block was used under ultrasound guidance. The appropriate point for the puncture was chosen by tracking the lateral side of the sternocleidomastoid muscle at the level of the Erb’s point. The needle was inserted perpendicularly to the skin and posteriorly to the sternocleidomastoid muscle, just below the superficial cervical fascia and the investing layer of the deep cervical fascia. About 20 mL of Ropivacaine (Naropin, Aspen Pharma Ltd, Dublin, Ireland) in a concentration of 375 μg/mL was injected within 5 min under ultrasound control.

Routine intraoperative monitoring included ECG, pulse oximetry, and non-invasive blood pressure monitoring. Patients were instructed after cross-clamping to keep pressing a whistling rubber duck that was placed on their hand contralateral to the surgery. The patient’s neurological status was monitored throughout the procedure by the anesthetist. The patient was considered SY if after cross-clamping any significant change developed in his/her consciousness, contralateral paresis, numbness of the muscles could be observed, or aphasia developed. In these cases, shunt insertion was indicated and performed by the surgeon.

Bilateral NIRS monitoring of the brain tissue was performed using the INVOS 5100C Cerebral Oximeter System (Somanetics Corporation, Troy, MI, USA) device throughout the procedure. Probes were placed on the forehead of the patients on both sides. Although the monitoring was continuous, only the following readings were taken into account for further analysis: (1) resting state, before regional block, (2) after block, before incision, (3) before cross-clamp (4) after cross-clamp, (5) 5 min after cross-clamp, (6) 10 min after cross-clamp, (7) after declamping, and (8) during the postoperative period (4–6 h).

In all the cases computed assisted tomography of the brain and CT angiography were performed before surgery. CT angiographies were later analyzed offline by three independent neuroradiologists who were unaware of the grouping status of the patients (SY or ASY after cross-clamping). Based on the CT angiographies these radiologists made a final decision in each case on whether the CoW can be considered functional or non-functional. An incomplete CoW was described if any anatomical or pathological condition was present that makes the CoW incomplete for collateral function (missing communicating arteries, occluded segments), and/or communicating artery diameters were below 0.5 mm. Categorization was based on former observations describing that 0.5 mm of the communicating arteries can be considered as the threshold diameter for collateral patency [15–17]. The neuroradiologists (I.B., E.N., Z.F.) independently assessed the CT angiographies and in those cases where discrepancies were found, categorization occurred based on a consensus after a common reassessment of the scans.

### 1.1 Statistical analysis

Mean and standard deviations are reported for all values. Categorical variables were compared with the appropriate chi-squared test. Repeated measure analysis of ANOVA was used for the comparison of the NIRS values at different phases of the procedure. Differences were considered statistically significant if the *p* value was <0.05.


**Ethical approval:** The research related to human use has been complied with all the relevant national regulations, institutional policies and in accordance the tenets of the Helsinki Declaration, and has been approved by the authors’ institutional review board or equivalent committee. The trial was registered in the ClinicalTrials.gov registry under the number NCT02665104. Investigations were approved by the institutional ethics board of the university (registration number: DE RKEB/IKEB:4364/2015).
**Informed consent:** Informed consent has been obtained from all individuals included in this study.

## 2 Results

One hundred and seven patients were included in the study. NIRS monitoring could be completed in all cases, whereas CT angiographies were available in 94 cases. The missing 13 patients were all ASY (= no symptoms during cross-clamping). The clinical characteristics of the patients are summarized in Table 1.

**Table 1:** Confounding factors of ASY and SY patients

	ASY after cross-clamp (*n* = 81)	SY after cross-clamp (*n* = 13)	*p*-value
Age (years)	67.8 ± 7.3	69.5 ± 4.6	0.09
Hypertension (Y/N)	66/15	10/3	0.69
Diabetes (Y/N)	22/59	5/8	0.40
Smoking (Y/N)	46/36	6/7	0.50
Coronary artery disease (Y/N)	36/45	8/5	0.25
Peripheral arterial disease (Y/N)	39/42	6/7	0.89
Stroke in history (Y/N)	40/41	7/6	0.76
Preoperative brain CT			
No ischemia	24	1	0.09
Lacunar infarction	39	5	0.51
Territorial infarction	31	7	0.28
Stroke during day 30	3	1	0.5
Contralateral hemodynamically significant ICA stenosis or occlusion	35	8	0.22

### 2.1 Correlation between completeness of the CoW and symptoms after cross-clamping

**Figure 1:**
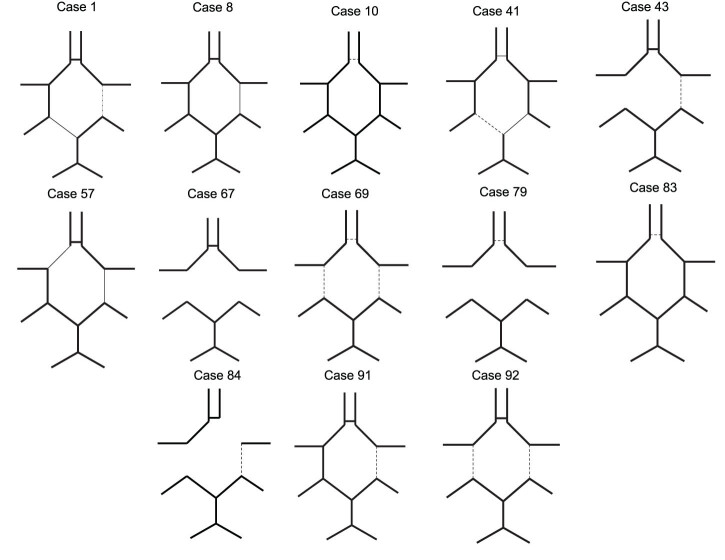
Graphical demonstration of CoW morphology in SY patients after cross-clamp with case numbers from the database. Thick solid lines indicate average diameter, thin solid lines indicate hypoplastic but functional segments, dotted lines indicate non-functional hypoplastic segments.

**Figure 2:**
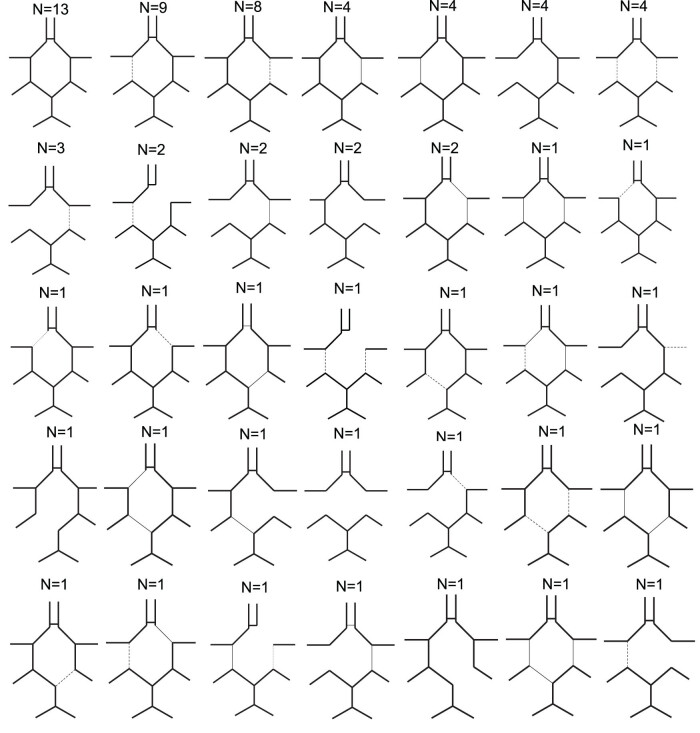
Graphical demonstration of CoW morphology in ASY patients after cross-clamp. *N* = indicates number of each variants found in the cohort. Thick solid lines indicate average diameter, thin solid lines indicate hypoplastic but functional segments, dotted lines indicate non-functional hypoplastic segments.

Based on CT angiography 67 incomplete circles were found in the entire cohort, 54 were ASY and 13 were SY after cross-clamping. Graphical demonstrations of the CoW morphologies are provided for SY patients in Figure 1 and for ASY patients in Figure 2. No complete CoW was found in those patients who showed symptoms after cross-clamping of the common carotid artery (*n* = 13). Among the ASY patients (*n* = 81) 54 were incomplete and 27 were complete circles. Statistically significant differences could be detected between the amount of incomplete and the complete circles between the ASY and SY groups (Chi-square: 6.08; *p* = 0.013).

### 2.2 Relationship between the functionality of communicating artery and symptoms after cross-clamping

The anterior communicating artery was missing or hypoplastic (<0.5 mm) in 5 of the 13 SY patients. There were no cases of the non-functional anterior communicating arteries in the ASY group (Chi-square: 32.9; *p* = 10^−8^). A missing or non-functional bilateral posterior communicating artery was observed in 9/13 SY and in 9/81 ASY patients (Chi-square: 24.4; *p* = 10^−7^). A non-functional posterior communicating artery on the side of surgical intervention was more frequently observed in SY patients (9/13) than in the ASY group (24/81, Chi-square: 7.71; *p* = 0.005) and the same was observed in non-functional posterior communicating artery contralateral to the endarterectomy (SY: 9/13; ASY: 28/81; Chi-square: 5.64; *p* = 0.017). Note that in the cohort only bilateral non-functional posterior communicating arteries were found among SY patients.

### 2.3 Brain tissue oxygen saturation during CEAs

**Figure 3:**
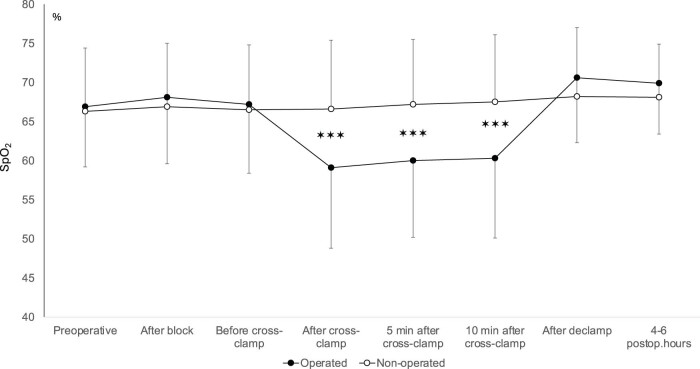
Regional cerebral oxygen saturation in ASY patients during different phases of CEA. Mean and standard deviations are shown. ✶✶✶ indicates *p* < 0.001 difference.

In ASY patients slight, but statistically significant differences could be detected in brain tissue oxygen saturation (rSO_2_) between operated and non-operated sides after cross-clamp. rSO_2_ was significantly lower after cross-clamping and this difference persisted until 10 min after cross-clamp placement. Of note, although statistically significant, the relative decrease of the rSO_2_ on the operated side did not reach 20% of the initial preoperative values. rSO_2_ values in ASY patients are depicted in Figure 3.

**Figure 4:**
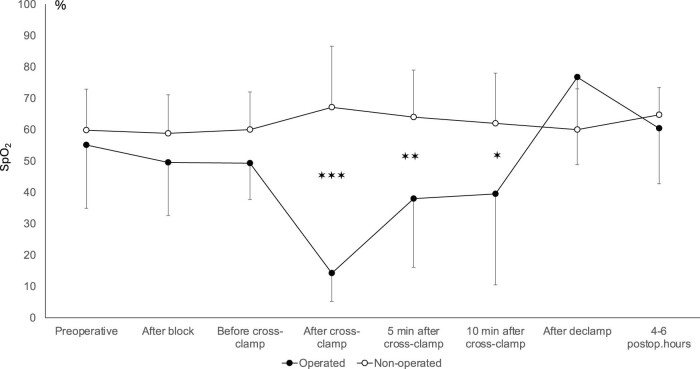
Regional cerebral oxygen saturation in SY patients during different phases of CEA. Mean and standard deviations are shown. ✶✶✶ indicates *p* < 0.001, ✶✶ *p* < 0.01 and ✶ *p* < 0.05 difference, respectively.

In SY patients rSO_2_ value gradually decreased after cross-clamping on the operated side and the difference between the two sides persisted until 10 min after cross-clamp. Although statistically not significant, an increased rSO_2_ value was observed on the operated side after cross-clamp, as a sign of ipsilateral hyperperfusion. No differences between rSO_2_ values between operated and non-operated sides could be found on readings recorded 4–6 h after the end of surgery. The results are shown in Figure 4.

## 3 Discussion

In this prospective observational study, we found that the non-functional CoW collaterals should be considered a risk factor of ischemic events after carotid cross-clamping during CEAs. Grouping of the patients undergoing regional anesthesia in the present study was based on the development of neurological symptoms referring to cerebral ischemia after cross-clamping of the common carotid artery. In all patients who experienced ischemic symptoms, absent or hypoplastic willisian collaterals were found and a significant decrease of the ipsilateral brain tissue oxygen saturation was detected.

Although strokes in the presence of hemodynamically significant internal carotid stenosis are considered to be primarily thromboembolic in origin [18], there are clinical observations supporting the importance of the willisian collaterals. In a follow-up study of patients without previous cerebrovascular disease, a combination of incomplete anterior and posterior collaterals increased the hazard ratio for ischemic stroke to 5,4-7 [9]. In an earlier report, Harrison and co-workers described that ischemic lesions of the CT scans are associated with the absence of intracranial collaterals in patients with internal carotid artery occlusions [19]. In patients with ischemic strokes, Hoksbergen et al. proved that incomplete or non-functional CoW is an independent risk factor for the event and the relationship is even stronger if a hemodynamically significant ipsilateral internal carotid artery lesion is present [7]. The prevalence of CoW variants was higher in stroke patients compared to controls in the study of De Caro et al. [20]. Additionally, in a recent report, an incomplete CoW has been shown to worsen the outcome of ischemic stroke patients [21]. Preoperative perfusion images, such as transcranial Doppler, single photon emission computed tomography, positron emission tomography, and arterial spin labeling MRI may help in the perioperative assessment of CEA. However, there is a lack of sufficient scientific evidence to confirm the benefits, necessitating further study [22].

There are basically two surgical attitudes regarding the use of shunts during CEAs: habitual shunters advocate the routine use of shunts whereas selective shunters perform shunt insertion only based on the results of various monitoring techniques (EEG,carotid stump pressure, transcranial Doppler, or somatosensory evoked potentials, if general anesthesia is applied) or based on intraoperative neurological symptoms (in CEAs performed in local anesthesia) [23]. The drawback of general anesthesia is the need of monitoring during the entire surgical procedure. However, many of these techniques have low sensitivity to detect intraoperative ischemia, their readings are influenced by the anesthetics [24] and therefore vascular surgeons cannot rely on their results while making decisions on shunting needs. Besides the fact that shunt insertion itself may also pose some intraoperative risks [3], based on a recent meta-analysis there are insufficient data to support either the selective or routine shunting strategy [25]. Banga and co-workers in their recent study proposed to obtain a preoperative angiographic assessment of the CoW collaterals for surgeons who routinely do not use shunts because they found a higher rate of postoperative strokes in those patients who were not shunted but had non-functional intracranial pathways [13]. This concept is also supported by a recent observation of Squizzato et al. who found that if the decision on shunt use is made in the intraoperative setting, it may elevate intraoperative stroke risk [3].

In the present study, we used clinical symptoms for classifying patients into SY and ASY groups, but the results of NIRS monitoring of the brain tissue oxygenation corresponded to the symptoms referring to cerebral ischemia after cross-clamping. When considering the appearance of neurological symptoms as comparators, a sensitivity of 76.9%, a specificity of 74.5%, a positive predictive value of 29.1%, and a negative predictive value of 95.9% were found for NIRS. These results correspond to those previously published by Moritz et al. [11] and are in line with the result of the meta-analysis by Duarte-Gamas who reported on a sensitivity of 72.0% and a specificity of 84.1% [26]. The moderate sensitivity and weak positive predictive value however again underline the necessity of other, more sensitive monitoring techniques during CEAs. From clinical point of view, the results of decreased cerebral oxygen saturation can be used for indicating shunt insertion with caution.

When it comes to the limitations of our study, we first have to mention the moderate number of included patients (*n* = 107). However, in this prospective study, the cooperation of at least three different disciplines was necessary to collect clinical and radiologic data. Additionally, offline analysis of the CT angiograms by three independent radiologists also necessitated extensive work. We believe, even this moderate number of cases was able to provide some hints that may be the basis of a prospective multicentric or synthesis study including a larger number of cases. We may be criticized for the diameter threshold used for defining functional and non-functional collaterals. In fact, previous studies mainly used 1 or 0.8 mm as the threshold for defining hypoplasia of the collateral vessels. However, these previous diameters were arbitrarily chosen and may have underestimated the non-functional collaterals. Several clinicopathological and model studies indicated that anterior and posterior communicating arteries can be considered non-functional if their narrowest diameter is below 0.5–0.6 mm [15–17]. Finally, in the present analysis, we mainly focused on the morphology of the CoW and did not take additional factors of positive awake tests into account, such as contralateral stenosis, hypertension, and diabetes. As there were no differences between these confounding factors between the ASY and SY groups, these factors may not have large impact on the present results.

In conclusion: incomplete or non-functional CoW may be considered a risk factor for ischemic events during CEAs. Further multicentric studies are warranted to prove whether preoperative assessment of the collateral capacity of the CoW may be helpful in decision-making about shunt use during CEA.
